# New insights into the karyotype evolution of the free-living flatworm *Macrostomum lignano* (Platyhelminthes, Turbellaria)

**DOI:** 10.1038/s41598-017-06498-0

**Published:** 2017-07-20

**Authors:** Kira S. Zadesenets, Lukas Schärer, Nikolay B. Rubtsov

**Affiliations:** 1 The Federal Research Center Institute of Cytology and Genetics SB RAS, Lavrentiev ave., 10, Novosibirsk, 630090 Russian Federation; 2 Evolutionary Biology, Zoological Institute, University of Basel, Basel, CH-4051 Switzerland; 3 Novosibirsk State University, Pirogova str., 2, Novosibirsk, 630090 Russian Federation

## Abstract

The free-living flatworm *Macrostomum lignano* is a model organism for evolutionary and developmental biology studies. Recently, an unusual karyotypic diversity was revealed in this species. Specifically, worms are either ‘normal’ 2n = 8, or they are aneuploid with one or two additional large chromosome(s) (i.e. 2n = 9 or 2n = 10, respectively). Aneuploid worms did not show visible behavioral or morphological abnormalities and were successful in reproduction. In this study, we generated microdissected DNA probes from chromosome 1 (further called MLI1), chromosome 2 (MLI2), and a pair of similar-sized smaller chromosomes (MLI3, MLI4). FISH using these probes revealed that MLI1 consists of contiguous regions homologous to MLI2-MLI4, suggesting that MLI1 arose due to the whole genome duplication and subsequent fusion of one full chromosome set into one large metacentric chromosome. Therefore, one presumably full haploid genome was packed into MLI1, leading to hidden tetraploidy in the *M. lignano* genome. The study of *Macrostomum* sp. 8 — a sibling species of *M. lignano* — revealed that it usually has one additional pair of large chromosomes (2n = 10) showing a high homology to MLI1, thus suggesting hidden hexaploidy in its genome. Possible evolutionary scenarios for the emergence of the *M. lignano* and *Macrostomum* sp. 8 genomes are discussed.

## Introduction

The genomes of many extant species show evidence of past whole genome duplications (WGDs). WGDs have occurred in many lineages, including amphibians, fishes, yeasts, flowering plants, and vertebrates, all of which are being studied by modern genomics^1, 2^. A WGD is usually followed by rediploidization as a result of gene divergence^3^. While the typical lifetime of duplicated genes has been estimated to be in the order of several millions of years^4, 5^, the WGDs in the vertebrate and teleost lineages took place mostly about 500 and 350 MYA (million years ago), respectively. The age of these WGDs therefore makes them unsuitable for the study of the first steps of genome evolution towards rediploidization^6^, and there are relatively few animal species that are known to have gone through WGDs recently. One of them is the African clawed frog *Xenopus laevis* (2n = 36), in which the last WGD dates back about 40 MYA^6^. Cross-species fluorescence *in situ* hybridization (FISH) using DNA probes generated from chromosomes of the only known diploid clawed frog species, *X. tropicalis* (2n = 20) revealed an allotetraploid — i.e. cross-species hybridization — origin of the *X. laevis* genome^7^. Another WGD was recently discovered in the genus *Cyprinus*, which dates back to about 8 MYA and which resulted in the genome of the common carp *Cyprinus carpio* ^8^. In contrast to other teleost species, which have undergone three rounds of WGD, *C. carpio* has undergone one additional round of species-specific WGD, which has resulted in a duplicated chromosome set (2n = 100)^9, 10^. So while relatively little is currently known about the early stages of post-WGD genome evolution in vertebrates, even less is known in invertebrates.

In general, WGD, as is true for other kinds of gene duplication events, leads to changes in the make-up of the whole genome and karyotype and thereby opens up possibilities for the evolution of new molecular functions, e.g. by facilitating neo- or subfunctionalization of genes and gene networks^11–13^. Moreover, the global changes in genome and karyotype reorganization, including subsequent chromosome rearrangements after WGD, may also lead to reproductive isolation from the ancestral form.

We have recently proposed that the clade containing the free-living flatworm *Macrostomum lignano* may have experienced a recent genome duplication^14^ as compared to other species in the genus *Macrostomum* ^14–17^. The usual karyotype of *M. lignano* is 2n = 8, with two large and six small metacentrics, while the karyotype of several other *Macrostomum* species (namely, *M. hystrix*, *M. tuba*, and *M. spirale*) is 2n = 6, with three pairs of small (sub)metacentric chromosomes, which are of similar size to the small metacentrics of *M. lignano* ^14^. Furthermore, some laboratory lines of *M. lignano* showed a high frequency of aneuploidy of the largest chromosome (further called MLI1), and aneuploid worms showed no visible morphological or reproductive abnormalities^14^.

Interestingly, *Macrostomum* sp. 8, a sibling species of *M. lignano* (T. Janssen and L. Schärer, unpublished data), has a karyotype that is similar to that of *M. lignano*, with suggested tetrasomy for its largest chromosome (2n = 10) being the most common karyotype, but with aneuploidy of chromosome 1 (2n = 9 and 2n = 11) also occurring with appreciable frequency^14^. In contrast to the aneuploidy described in *M. lignano* and *Macrostomum* sp. 8, whole-chromosome aneuploidy in many taxa (including plants, invertebrates, mammals) often results in severe developmental disorders, diseases, and lethality^18–20^. This prompted the questions about how to interpret these considerable levels of aneuploidy in these two *Macrostomum* species. Our previous results led us to suggest that the karyotype in *M. lignano* may represent a form of hidden polyploidy^14^.

In the current study, we test the proposition that the *M. lignano* genome has evolved from an ancestral genome following a WGD event and that a subsequent fusion of one full set of chromosomes has then led to the formation of the large metacentric MLI1 chromosome. If so, the observed 2n = 8 karyotype would represent a tetraploid, and the observed 2n = 9 and 2n = 10 aneuploids could therefore be considered as hidden penta- and hexaploids, showing no genetic imbalance. To test this we explore the genome structure in both *M. lignano* and its sibling species *Macrostomum* sp. 8.

## Results

### Establishing the DV1/10 subline of *M. lignano*

After 3 months of culture maintenance (4 generations), 100 DV1/10 worms were randomly selected and karyotyped. Most of them (97 specimens) had the expected chromosome number 2n = 10. Of the other three, one had 2n = 9 (3 large metacentrics and 6 small metacentrics), one had 2n = 11 (5 large metacentrics and 6 small metacentrics), and one had 2n = 15 (6 large metacentrics and 9 small metacentrics). After one additional year of culture maintenance (20 generations), the DV1/10 line was karyotyped again. In a selection of 100 newly karyotyped worms, 96 had the expected 2n = 10 karyotype. Of the other four, one was 2n = 10 (with 4 large and 5 small of the usual metacentrics, plus one small submetacentric chromosome), two were 2n = 11 (5 large metacentrics and 6 small metacentrics), and one was 2n = 14 (6 large metacentrics and 8 small metacentrics).

### Generation of chromosome-specific microdissected DNA probes

**Figure 1:**
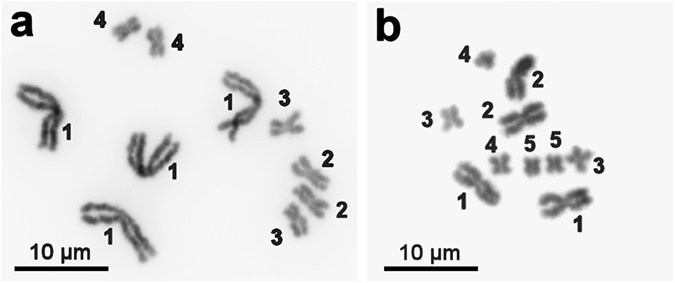
Metaphase spreads of *M. lignano* (**a**) and *Macrostomum* sp. 8 (**b**). (**a**) A metaphase spread of the *M. lignano* line DV1/10 with 2n = 10 (4 m + 2 m + 2 m + 2 m). The modified technique used here allowed us to obtain high-quality chromosome spreads and to also reliably identify chromosome 2 (MLI2). (**b**) A metaphase spread of *Macrostomum* sp. 8 with 2n = 10 (4 m + 2 m + 2 m + 2 m). The chromosomes are designated by Arabic numerals.

After metaphase chromosome preparation and Giemsa staining, chromosome MLI1 could always be clearly distinguished from the other chromosomes based on its size (Fig. 1), whereas the three pairs of smaller chromosomes often appeared similar in morphology and size. According to an earlier morphometric analysis of metaphase chromosomes, the average lengths of chromosomes MLI2, MLI3, and MLI4 were 2.74 ± 0.27 µm, 2.49 ± 0.025 µm, and 2.24 ± 0.29 µm, respectively^14^. However, on the high-quality prometaphase and early-metaphase plates we obtained in the current study, MLI2 could also be reliably identified, while MLI3 and MLI4 could not be reliably distinguished (Fig. 1a). The optimized technique for chromosome preparation allowed identification and collection of chromosomal material belonging to definite chromosomes (at least, MLI1 and MLI2).

### Fluorescence *in situ* hybridization (FISH) in *Macrostomum lignano*

**Figure 2:**
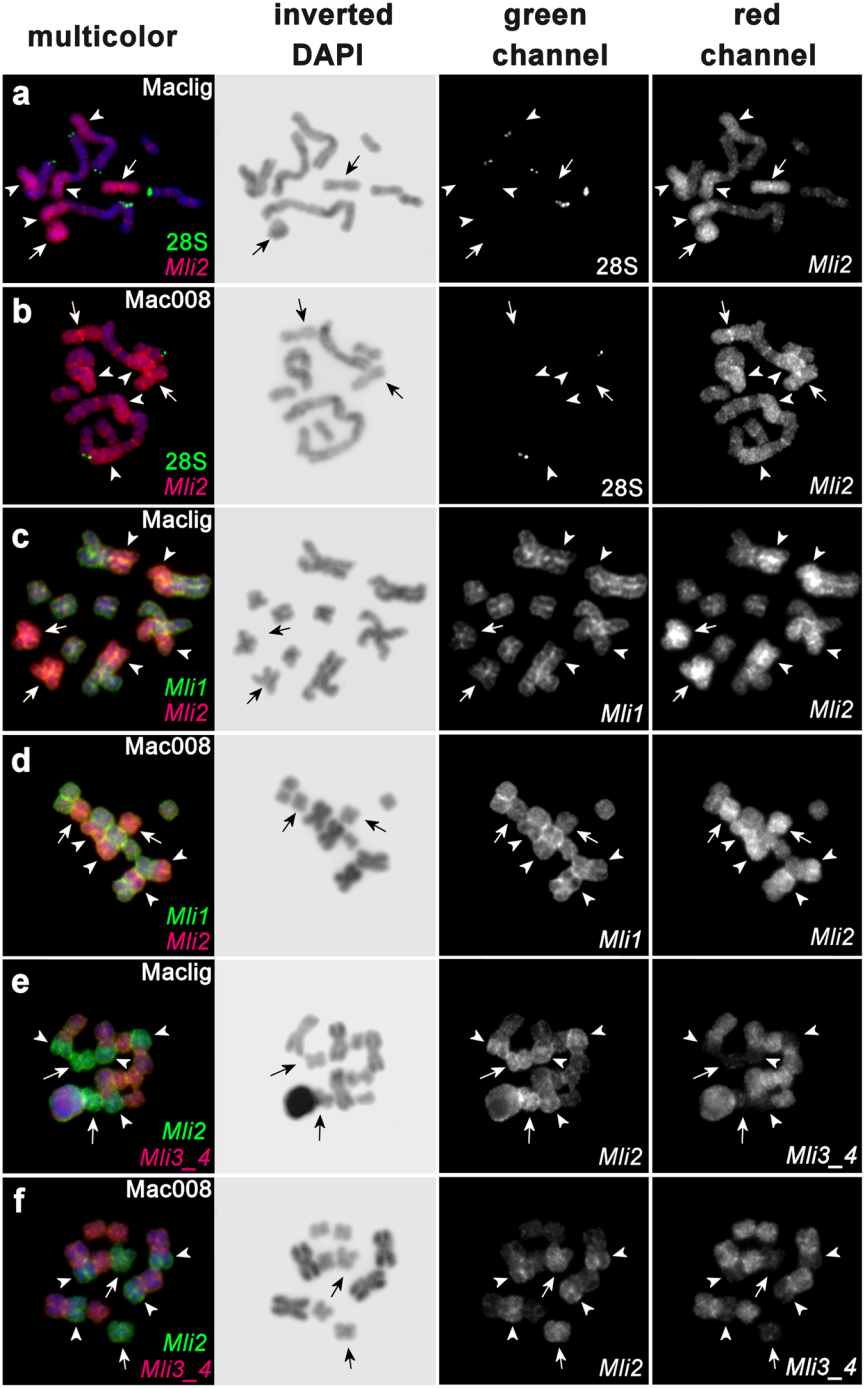
Two-color fluorescence *in situ* hybridization (FISH) of the *M. lignano* line DV1/10 (**a**,**c**,**e**) and *Macrostomum* sp. 8 (**b**,**d**,**e**) chromosomes using microdissected DNA probes and a 28S rDNA probe from *M. lignano* (counterstained with DAPI). (**a**,**b**) 28S rDNA (green) and *Mli2* (red); (**c**,**d**) *Mli1* (green), *Mli2* (red), co-localized FISH signal (orange); (**e**,**f**) *Mli2* (green) and *Mli3_4* (red), co-localized FISH signal (orange). Inverted DAPI images are to the right of the FISH images. Chromosome MLI2 in (**a**,**c**,**e**) (chromosome 3 of *Macrostomum* sp. 8 in (**b**,**d**,**f**) is indicated by arrows. *Mli2*-painted regions of MLI1 (chromosome 1 and 2 of *Macrostomum* sp. 8, respectively) are indicated by arrowheads.

Following CISS-hybridization, the DNA probes *Mli1*, *Mli2*, and *Mli3_4* all painted intensively the pericentromeric regions, suggesting that all these regions contain clusters of homologous repeats (Fig. 2a). With respect to the chromosome arms, FISH with probe *Mli2* painted intensively chromosome MLI2 and a contiguous region on the long arms of all four MLI1 copies (Fig. 2a,c,e), while probe *Mli1* painted all chromosomes (as previously observed in ref. 14), leading to double-labeled regions (Fig. 2c,e). Interestingly, all MLI1 copies were painted identically (see below for details on the 28S rDNA probe). The background FISH signal in the other chromosomal regions presumably comes from labeled interspersed DNA repeats and suggests insufficient suppression of repetitive DNA hybridization.

Morphometric analysis of chromosome MLI1 showed that in all the chromosome spreads analyzed, the MLI1 arm containing the painted region was longer than the other arm of this chromosome (Table 1). Thus, the FISH painting of *M. lignano* chromosomes with probe *Mli2* revealed in the q-arm of MLI1 (MLI1q) a region homologous to a very substantial part of MLI2 (Fig. 2a,c,e). Morphometry showed that MLI2 was somewhat longer than the *Mli2* painted region in MLI1q (Table 1). Furthermore, no intensive signal that was similar to the FISH signal in the pericentromeric region of MLI2 was observed within the painted region of MLI1q. We also note that no cluster of telomeric repeats was previously observed at the proximal end of the painted region (i.e. there were no interstitial telomeric sequences in MLI1, see ref. 14). This suggests that both clusters of pericentromeric and telomeric repeats were probably lost from the painted region of MLI1q.

**Table 1:** Morphometry of the MLI1 arms with respect to the regions painted with *Mli2*. The measurements were performed on 10 metaphase spreads and the reported values represent means ± 1 SD.

Length of the MLI1 arms (µm)		Length of the *Mli2* regions painted (µm)	
MLI1p without a painted region	MLI1q with the painted region	on MLI1	on MLI2
2.945 ± 0.705	3.584 ± 0.724	2.44 ± 0.49	3.44 ± 0.477

FISH with *Mli3_4* revealed specific signals in two pairs of small chromosomes (identified as MLI3 and MLI4; Fig. 2e, red) and in the region of MLI1 that remained unpainted with *Mli2* (Fig. 2e, green). Like FISH with *Mli2*, FISH with *Mli3_4* produced some signal in all pericentromeric regions (Fig. 2e, orange). No additional regions with increased FISH signals were revealed in the regions painted with *Mli3_4*. Simultaneous FISH with *Mli2* and *Mli3_4* painted chromosome MLI1 completely (Fig. 2e), with some colocalization in the pericentromeric regions (Fig. 2e, orange).

FISH with the 28S rDNA (green) and the *Mli2* (red) probes revealed a 28S rDNA cluster at the ends of the p-arm of MLI1 (Fig. 2a). Moreover, on prometaphase chromosomes we could identify a cluster of 28S rDNA at the end of the q-arm of MLI3 (see also ref. 14), which might thus allow us to distinguish this chromosome from MLI4.

### Fluorescence *in situ* hybridization in *Macrostomum* sp. 8

Identical FISH experiments with the same combinations of chromosome-specific DNA probes and labeled 28S rDNA probe from *M. lignano* were performed on chromosomes of *Macrostomum* sp. 8. The painting patterns obtained (Fig. 2b,d,f) were almost identical to those observed in *M. lignano* line DV1/10 chromosomes. FISH with labeled rDNA detected only one cluster of 28S rDNA on *Macrostomum* sp. 8 chromosomes, namely on chromosome 4 (Fig. 2b), and confirmed the previously reported localization of a cluster of 28S rDNA in one of the small chromosomes^14^. MLI1 appeared to be homologous to *Macrostomum* sp. 8 chromosomes 1 and 2; MLI2, to *Macrostomum* sp. 8 chromosome 3; MLI3, to *Macrostomum* sp. 8 chromosome 4 (i.e. the chromosomes with the 28S rDNA cluster); and MLI4, to *Macrostomum* sp. 8 chromosome 5 (Fig. 1a,b).

## Discussion

Generation of microdissected DNA probes followed by modified CISS-hybridization provides compelling evidence in support of a hidden tetraploidy in the usual 2n = 8 karyotype of *M. lignano*. Our results suggest that post-WGD chromosome evolution has fused almost one full haploid set of chromosomes into the largest chromosome of *M. lignano*, MLI1, while two clusters each of pericentromeric and telomeric repeats have apparently been lost from MLI1 due to chromosomal rearrangements, since our FISH experiments revealed no remnants of telomeres or centromeres in either MLI1p or MLI1q^14^. The region of chromosome MLI1 painted with the *Mli2* probe appeared to be somewhat shorter than chromosome MLI2, and we think that the loss of the repeat clusters alone likely cannot fully explain this shortening. Conversely, FISH of the *Macrostomum* metaphase chromosomes with the *Mli1* probe revealed no region in the small metacentrics left unpainted. These results allow us to conclude that no large chromosomal regions were lost during the chromosomal rearrangements that led to MLI1 formation (given the observed chromosome condensation levels and FISH conditions used here, we expect that we could have detected such regions if they were larger than about 3 Mb). The search for smaller lost regions will require additional techniques, such as high-throughput sequencing and FISH with unique DNA fragments, but this goes beyond the scope of the current study.

When considering the difference in length between MLI2 and the *Mli2*-painted region of MLI1q, we should take into account the fact that we have previously identified a higher level of condensation of MLI1 compared to the condensation observed in the other *M. lignano* chromosomes in metaphase^14^. For this reason, we only used prometaphase and early metaphase chromosomes for morphometry in the present study. Nevertheless, it is still possible that the respective lengths of MLI2 and its homologous region in MLI1q, as inferred by morphometry, were affected by the level of condensation in chromosomes MLI1 and MLI2. Therefore, the question about putatively lost chromosomal regions, other than the above-mentioned clusters of pericentromeric and telomeric repeats, must remain open at this stage.

**Figure 3:**
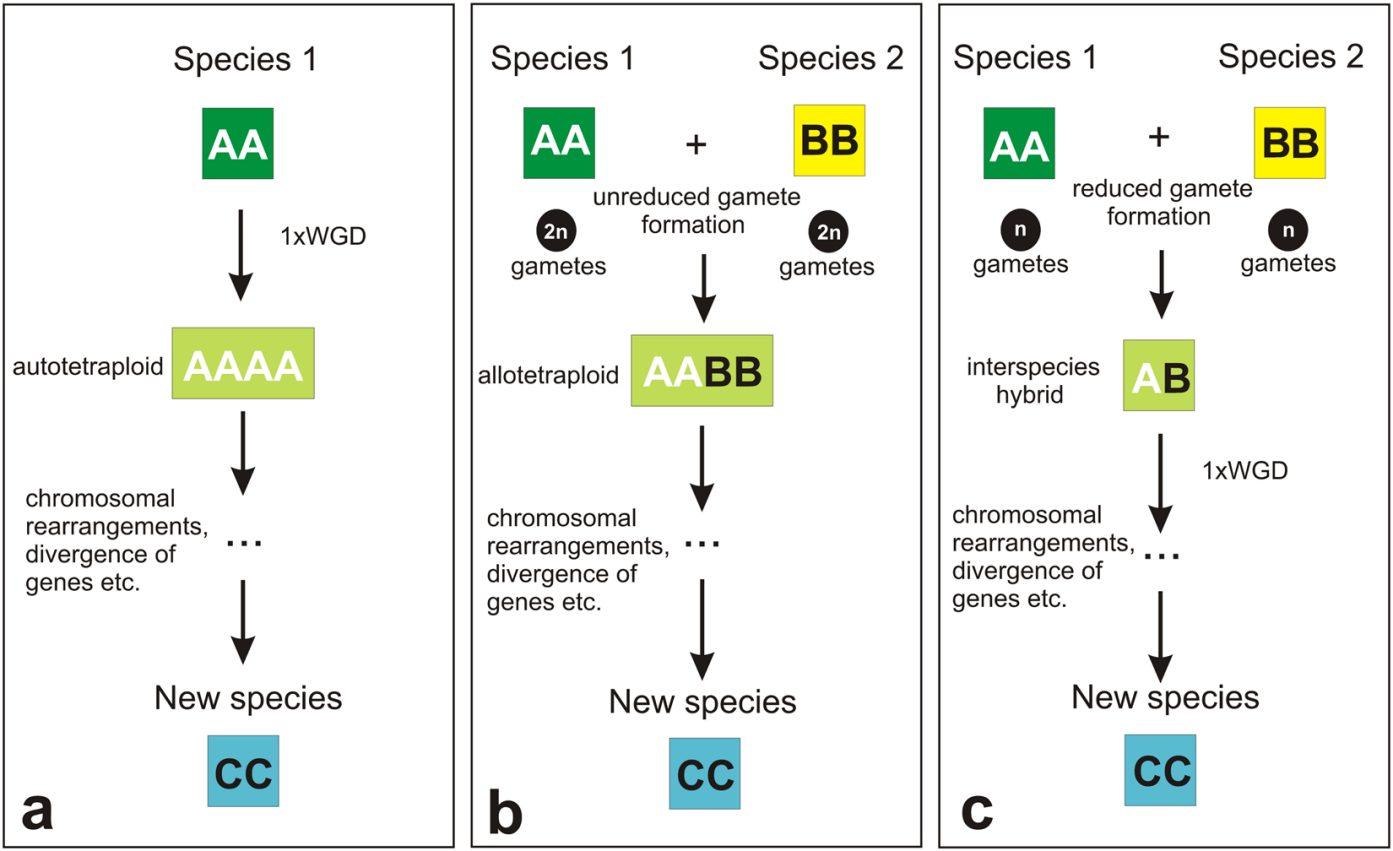
Possible scenarios for autoploid (**a**) and allopolyploid (**b**,**c**) formation of the *M. lignano* genome. (**a**) Autotetraploid (2n = AAAA) formation from a diploid species (2n = AA); (**b**) Hybrids (2n = AABB) formed from crosses between two closely related diploid species 1 (2n = AA) and 2 (2n = BB) with polyploidy through unreduced gamete formation, (**c**) Hybrids (2n = AB) formed from hybridization between species 1 (2n = AA) and 2 (2n = BB) without polyploidy, but followed by one WGD.

The data obtained here are in a good agreement with the idea that the *M. lignano* genome arose through a single WGD, followed by chromosomal rearrangements, which fused one full haploid set of chromosomes in the ancestral karyotype into one large chromosome. We propose three possible scenarios for *M. lignano* genome evolution (Fig. 3). Scenario A (Fig. 3a) includes a direct WGD of the ancestral genome at the first stage. The second stage includes global chromosomal rearrangements, such as bringing one full haploid set of chromosomes in the ancestral genome into one large chromosome. These chromosomal rearrangements may have solved, at least in part, the meiotic problems that one could expect to occur in tetraploids. With FISH, we did not reveal remnants of ancestral pericentromeric or telomeric DNA repeats at the ancestral chromosome fusion sites. The loss of telomeric regions may have occurred immediately during the chromosome fusion event at stage two or later, while the centromeres with pericentromeric repeats were probably lost in the following stages of evolution^21, 22^.

Under Scenario B, individuals of two ancestral species both produce unreduced (diploid) gametes due to meiotic failure, and the fusion of two diploid gametes results in allotetraploidy (Fig. 3b). While this scenario does not appear very likely (since it requires the likely rare combination of unreduced gametes in both species plus hybridization), we here nevertheless include it as a theoretical possibility. Under Scenario C (Fig. 3c), the first stage includes interspecies hybridization (or allopolyploidization) between two closely related ancestral species. A direct WGD then occurs in the hybrid genome, which results in allotetraploidy (Fig. 3c). Evidence exists that interspecific hybrids themselves very commonly produce higher frequencies of unreduced gametes than their progenitor species^23^. The next stages of *M. lignano* genome evolution under Scenarios B and C then follow as under Scenario A. At the current point of our study, it is impossible to determine whether allopolyploidy occurred through a WGD, followed by long-term evolution or whether it was part of a speciation process through interspecific hybridization.

Studies on genome evolution have over the last decades shown that interspecific hybridization is a much more important mechanism of speciation than was previously thought^23–27^. Moreover, this mechanism is even more important for the generation of genomic diversity in plants^24, 28, 29^. To date, only few studies have proposed that many WGDs in both plants and animals may have resulted from unreduced gamete formation^23, 27^.

It has been proposed that WGDs are usually followed by massive and rapid gene loss and structural rearrangements^30–32^. However, traces of duplication events may remain for long time periods and can be detected by complex comparative genomic analysis (e.g. identification of inter- and intra-genomic collinearity; phylogenetic reconstruction of gene family evolution; analysis of *K*_S_ age distribution^33^). However, phylogenetic lineages exist in which no massive gene loss has occurred since the WGD. For example, *C. carpio* has undergone a fourth WGD only about 8 MYA ago, and most of the duplicated ancestral genes remain present in the *C. carpio* genome^34^. Moreover, about 25% of the recently duplicated genes that were analyzed showed some level of functional divergence, and among these cases neo- and sub-functionalization appear to be the main outcomes^8^. As far as the *M. lignano* genome is concerned, further studies require high-quality genome assemblies and comparative analyses of the genomes of species that are closely related to *M. lignano*.

The most striking finding of our recent studies, however, was that *M. lignano* and *Macrostomum* sp. 8 show unexpectedly high levels of intraspecific karyotype diversity. Primarily, many cases of tri- and tetrasomy on chromosome 1 were revealed (and also some pentasomy in *Macrostomum* sp. 8), while we also observed some rare cases of gain or loss of small chromosomes in both species (unpublished data). In our studies we probably mainly karyotyped somatic cells, since the regeneration blastema that are induced by the amputation are likely driven by somatic stem cells^35^, but in previously performed crossing experiments, controlled crosses between euploid and aneuploid worms the resulting offspring clearly suggested aneuploid gamete formation^14^.

It should be noted that in many types of organisms whole-chromosome aneuploidy often leads to severe detrimental effects, such as serious malformations, diseases, and lethality^19, 36^. For example, studies of aneuploidy in model species from different taxa (plants, invertebrates, mammals) have revealed that aneuploidy has severe effects on development and growth^18, 37–39^, and in humans, aneuploidy is associated with abnormalities in cell function (including cancer) and organismal development^40^.

In the current study, we provide evidence that the large chromosome, MLI1, in the *M. lignano* karyotype has arisen due to a WGD and subsequent fusion of one full haploid chromosome set into this large metacentric chromosome, leading to the usual 2n = 8 karyotype of this species. Therefore, what we formerly considered to be cases of aneuploidy of MLI1, namely the 2n = 9 and 2n = 10 karyotypes, now instead appear to be cases of hidden polyploidy. Specifically, a straight ploidy series, with the 2n = 8, 2n = 9, and 2n = 10 karyotypes representing, respectively, tetra-, penta-, and hexaploids, could explain why the individuals of *M. lignano* (and also *Macrostomum* sp. 8) having additional copies of the large chromosome do not show severe abnormalities and even produce viable offspring (yet to be shown for *Macrostomum* sp. 8 in ref. 14).

So while trisomy and tetrasomy for the largest *M. lignano* chromosome, MLI1, would not lead to gene dosage imbalance, gene dosage might of course still be disturbed by aneuploidy of one of the small chromosomes. Among hundreds of karyotyped worms we found only a few specimens with aneuploidy for one of the small chromosomes, and interestingly, also these worms did not show any significant abnormalities (although they were too rare to be included in crossing experiments to test their siring ability). We speculate that some level of tolerance to aneuploidy for small chromosomes could derive from the presence of the hidden tetraploidy in the *M. lignano* genome, since any gene dosage imbalance resulting from small chromosome aneuploidy might be less harmful in the tetraploid background compared to normal diploids.

Aneuploidy can potentially result from errors in both meiosis and mitosis. With respect to the latter, for many species there exist data on mosaic individuals that are characterized by abnormal karyotypes of some of their somatic cells^40^, including, for example, cancer cells that often contain numerous chromosome rearrangements. To date we have karyotyped several hundreds of individual worms but have never found clear cases of mosaic individuals. Instead, the karyotypes of nearly all analyzed cells belonging to the one specimen were always identical, indicating a reliable and precise mechanism of mitosis in these species. With respect to the former, karyotypic abnormalities could of course also arise as result of mistakes occurring during meiosis^20^, although it has been argued that in species with sexual reproduction, meiosis and syngamy may actually represent a barrier to the spread of abnormal karyotype formation^41^. In some previously performed crossing experiments, we indeed observed a small fraction of offspring with karyotypes that were unexpected when considering their parental karyotypes (unpublished data), suggesting that meiotic errors could potentially be a reason for the appearance of worms with abnormal karyotypes. The extended homologous regions in the small and large chromosomes could lead to problems during meiotic chromosome conjugation. However, the question about the origin of worms having abnormal karyotypes has to remain open for now and will require more detailed investigations of meiosis and karyotype inheritance patterns.

The instability of the *M. lignano* and *Macrostomum* sp. 8 karyotypes among worms from our laboratory cultures and natural populations also renders plausible the formation of the *Macrostomum* sp. 8 genome through aneuploidy in *M. lignano* ^14^. The only striking karyotypic difference between *Macrostomum* sp. 8 and *M. lignano* line DV1/10 is the absence of the 28S rDNA repeats in the largest chromosome of *Macrostomum* sp. 8. The results obtained in the current studies may thus suggest that the *Macrostomum* sp. 8 genome was derived from the *M*. *lignano* genome in a way similar to how the *M. lignano* line DV1/10 genome was derived from the more ‘normal’ 2n = 8 *M*. *lignano* genome. This would suggest that the ‘normal’ 2n = 10 karyotype of *Macrostomum* sp. 8 is a hidden hexapoid—similar to *M. lignano* with 2n = 10—and that we can therefore expect exciting results from comparative genomics of *M. lignano* and *Macrostomum* sp. 8.

## Conclusions

The previously documented karyotype diversity in *M. lignano* and *Macrostomum* sp. 8, which is mainly represented by aneuploidies of the largest chromosome, prompted us to explore the organization of this chromosome in both *M. lignano* and *Macrostomum* sp. 8 using chromosome-specific microdissected DNA probes. Our results provide evidence for hidden tetra- and hexaploidy in the genomes of the ‘normal’ 2n = 8 and 2n = 10 karyotypes of *M. lignano* and *Macrostomum* sp. 8, respectively. Moreover, *Macrostomum* sp. 8 may have recently arisen from *M. lignano* or a closely related ancestor due to tetrasomy of the largest chromosome. As a result of chromosomal rearrangements accompanying the formation of MLI1 in *M. lignano*, nonfunctional ancestral telomeres and centromeres were apparently lost. It appears possible that that hidden tetraploidy in the *M. lignano* genome arose due to a WGD and/or an interspecific hybridization event between closely related *Macrostomum* species. Clarification of the mechanisms underlying genome evolution in *Macrostomum* species now requires further studies, including comparative genomics of species closely related to *M. lignano* and high-throughput sequencing of microdissected DNA libraries derived from individual chromosomes.

## Methods

### Study organisms

Members of two closely related species of the free-living flatworm genus *Macrostomum*, *M. lignano* and *Macrostomum* sp. 8, were maintained under standard laboratory conditions^42, 43^. The *M. lignano* inbred line DV1 has been widely used in a range of studies^14, 44–47^. It was created via full-sib and half-sib inbreeding for 24 generations, and has since been kept at small population sizes to maintain a high level of homozygosity^44^. It is important to mention that we uncovered a high frequency of aneuploids, and in a few cases also other numerical and structural chromosome abnormalities, within this inbred DV1 line^14^. The *Macrostomum* sp. 8 worms were cultivated as an outbred culture (i.e. starting every generation with 100 hatchlings to maintain genetic diversity) initiated from about 90 field-collected individuals (see also Table 1 in ref. 14).

### Establishing the DV1/10 subline of *M. lignano*

Given the above-mentioned karyotype variability observed within the DV1 inbred line^14^, we here aimed at establishing a line with a pure-breeding and thus more predictable karyotype. Earlier results suggested that it would likely be difficult to establish a pure-breeding 2n = 8 line, as 2n = 8 individuals were consistently underrepresented in that line, possibly as a result of a maintained polymorphism (i.e. selection against certain homozygous combinations of the MLI1 chromosomes^14^). We therefore instead aimed at establishing a pure-breeding 2n = 10 line (further called DV1/10) initiated from two worms that were selected from among a range of karyotyped specimens^14^ and which had a 2n = 10 karyotype and tetrasomy of chromosome MLI1.

### Metaphase chromosome preparation

Chromosome spreads were prepared using the cell suspension method in Carnoy’s fixative (methanol: glacial acetic acid, 3:1) as described previously, with some modifications^14, 48^. The hypotonic treatment was considerably prolonged and was performed in hypotonic 0.56% KCl solution for 2 hours at RT. For karyotyping and FISH experiments, the suspension was dropped onto cold wet microscope slides (76 mm × 26 mm, 1 mm thick), and for metaphase microdissection, the suspension was dropped onto clean cold wet cover slips (60 mm × 24 mm, 0.17 mm thick).

### Generation of chromosome-specific microdissected DNA probes

Chromosome microdissection was carried out as previously described^49^. Initial DNA amplification of the collected chromosomes was performed using a GenomePlex Single Cell Whole Genome Amplification Kit (WGA4) (Sigma-Aldrich) according to the manufacturer’s protocol. Microdissected DNA probes *Mli1* and *Mli2* were generated, respectively, from 15 copies of chromosome MLI1 (the largest chromosome) and MLI2 (the largest among the small chromosomes) of *M. lignano* (Fig. 1a). Moreover, microdissected DNA probe *Mli3_4* was generated from 15 copies of chromosomes MLI3 and MLI4 (note that these two chromosomes are too similar in size to be reliably distinguished on chromosome spreads; see also Results). The PCR products were labeled with Flu- or TAMRA-dUTP (Genetyx, Novosibirsk) in additional 20 PCR cycles using WGA3 kit (Sigma-Aldrich).

### Fluorescence *in situ* hybridization (FISH)

FISH with 28S rDNA probe was used as a quality control of *in situ* hybridization. The 28S rDNA probe was generated and hybridized on metaphase chromosomes as previously described^14^. FISH with microdissected DNA probes was performed on metaphase chromosomes of *M*. *lignano* and *Macrostomum* sp. 8 with salmon sperm DNA as a DNA carrier, as previously described^14^, with a minor modification to include -chromosome *in situ* suppression (CISS) - hybridization. Specifically, a 10× excess of unlabelled PCR product generated from genomic DNA of *M. lignano* was added to the DNA probe mix to decrease the fluorescent signal coming from labeled DNA repeats. Chromosome slides and DNA probes were denatured separately. After a denaturation step at 75 °C in 70% formamide/2× SSC for 3 min, the slides were dehydrated through a pre-cooled ethanol series (70%, 80% and 96%) and then left for air drying. The DNA probe mix was denatured at 96 °C for 3 min and incubated at 37 °C for 1 h (for pre-annealing of repetitive DNA). The remaining steps were performed according to the standard procedure^48^. After FISH, chromosomes were counterstained with DAPI dissolved in Vectashield antifade solution (Vector Laboratories, USA).

### Microscopic analysis

Microscopic images of chromosomes were captured and analyzed using a CCD-camera installed on an Axioplan 2 imaging microscope equipped with filtercubes #49, #10, and #15 (ZEISS, Germany) and using the ISIS4 software package (MetaSystems GmbH, Germany) at the Inter-institutional Shared Center for Microscopic Analysis of Biological Objects (Institute of Cytology and Genetics SB RAS, Novosibirsk, Russian Federation).
